# Using Caregiver Stories to Better Understand Burden and Assess Need

**DOI:** 10.1093/geroni/igaf122.1086

**Published:** 2025-12-31

**Authors:** Aileen McGrory, Jacqueline Boudreau, Lynette Kelley, Marianne Desir, Eileen Dryden

**Affiliations:** Veterans Health Administration, Bedford, Massachusetts, United States; Veterans Health Administration, Bedford, Massachusetts, United States; Veterans Health Administration, Aurora, Colorado, United States; Veterans Health Administration, Miami, Florida, United States; Veterans Health Administration, Bedford, Massachusetts, United States

## Abstract

While informal caregivers provide crucial care to older Veterans, they often experience high levels of personal stress and difficulties accessing support. Understanding caregiver burden is crucial to improving caregiver support, since recognition of burden is a first step in connecting the caregiver to resources. Many studies on caregiver burden focus on defining and measuring burden and its relationship with other factors, but the literature lacks consensus as to which factors are most correlated with burden. Given this divided literature, a closer qualitative look at caregivers’ experiences is warranted. In our sample of 30 caregivers of rurally located older Veterans, we found that Zarit Burden scores were closely aligned with interview data about caregiver experience. Other commonly used measures that we collected, including ADLs and IADLs, were not as closely associated with Zarit Burden score. Instead, caregiving difficulty among those with high Zarit Burden scores aligned with experiential elements including 1) concerns about the caregiver and care recipient’s relationship, 2) perceived lack of access to conversations about care, 3) isolation, and 4) focus on the future. Although objective burden is often easier to collect and quantify using measurements of caregiving tasks and time commitment, those methods exclude elements that shape caregiver experience but are only apparent through a more thorough understanding of caregivers’ stories. Including individual experience and subjective burden while assessing overall caregiver burden, for instance by incorporating brief caregiver “interviews,” may allow more insight into caregiver needs and help improve support, and thus care and outcomes of older patients.

